# Recent advances in molecular mechanisms of acute kidney injury in patients with diabetes mellitus

**DOI:** 10.3389/fendo.2022.903970

**Published:** 2023-01-05

**Authors:** Barbara Infante, Francesca Conserva, Paola Pontrelli, Serena Leo, Alessandra Stasi, Marco Fiorentino, Dario Troise, Andrea dello Strologo, Carlo Alfieri, Loreto Gesualdo, Giuseppe Castellano, Giovanni Stallone

**Affiliations:** ^1^ Department of Medical and Surgical Sciences, University of Foggia, Foggia, Italy; ^2^ Department of Emergency and Organ Transplantation, University of Bari Aldo Moro, Bari, Italy; ^3^ Nephrology, Dialysis and Renal Transplant Unit, Department of Clinical Sciences and Community Health, University of Milan, Fondazione IRCCS Cà Granda Ospedale Maggiore Policlinico, Milan, Italy

**Keywords:** acute kidney injury, diabetes mellitus, biomarkers, risk factors, chronic kidney diease

## Abstract

Several insults can lead to acute kidney injury (AKI) in native kidney and transplant patients, with diabetes critically contributing as pivotal risk factor. High glucose per se can disrupt several signaling pathways within the kidney that, if not restored, can favor the instauration of mechanisms of maladaptive repair, altering kidney homeostasis and proper function. Diabetic kidneys frequently show reduced oxygenation, vascular damage and enhanced inflammatory response, features that increase the kidney vulnerability to hypoxia. Importantly, epidemiologic data shows that previous episodes of AKI increase susceptibility to diabetic kidney disease (DKD), and that patients with DKD and history of AKI have a generally worse prognosis compared to DKD patients without AKI; it is therefore crucial to monitor diabetic patients for AKI. In the present review, we will describe the causes that contribute to increased susceptibility to AKI in diabetes, with focus on the molecular mechanisms that occur during hyperglycemia and how these mechanisms expose the different types of resident renal cells to be more vulnerable to maladaptive repair during AKI (contrast- and drug-induced AKI). Finally, we will review the list of the existing candidate biomarkers of diagnosis and prognosis of AKI in patients with diabetes.

## 1 Introduction

Epidemiological data points to diabetic kidney disease (DKD) as the main cause of end-stage renal disease (ESRD) worldwide and one third of patients with diabetes develop renal complications (1). Globally, kidney disease was responsible for approximately 1.2 million deaths in 2017 (2) and despite a recent decrease in mortality for ESRD in high-income countries, largely due to improved screening policies and novel drug regimens for the management of diabetes, the incidence of DKD has now risen among low- and middle-income countries (3). The economic burden of ESRD is extensive, considering the need for renal replacement therapy (RRT) and renal transplantation of affected individuals. Along, DKD is a major risk factor for several micro and macrovascular complications including diabetic retinopathy, diabetic neuropathy, coronary artery, cerebrovascular and peripheral artery disease (4). Finally, it has been largely shown that the physiological ageing of the kidney, characteristic of the elderly population, exposes this category of patients to a higher risk of AKI, especially when diabetes is associated to the aforementioned complications (5).

The importance of a correct classification of DKD has been underestimated over the past. Although renal biopsy is considered an invasive procedure, the existence of non-proteinuric DKD along with the presence of different patterns of renal damage in patients with diabetes may have led to poor clinical decision (6). It has been observed that only 40% of patients with DKD show the histological signs of Diabetic Nephropathy (DN), which include glomerular basement membrane (GBM) thickening, mesangial cell proliferation, nodular glomerular sclerosis (the Kimmelstiel-Wilson lesion) and tubulointerstitial fibrosis. The remaining fraction of patients show histological lesions that are a consequence of non-diabetic renal disease (NDRD) or of the concomitant presence of DN and NDRD lesions, thus the clinical picture can be complex and needs to be fully characterized for optimal treatment (7). It has also been shown that Diabetes Mellitus (DM) confers increased susceptibility for Acute Kidney injury (AKI), irrespective of age, proteinuria, hypertension and other comorbidities (8).

According to Kidney Disease Improving Global Outcomes (KDIGO) guidelines, stage I AKI is defined as an increase in SCr by >0.3 mg/dl within 48 h; or an increase in SCr to ≧̸1.5 times baseline occurred within the prior 7 days; or a urine volume <0.5 mL/kg/h for 6 hours. Thus AKI patients are monitored through measurements of SCr and urine output (9). Different sorts of insults can lead to AKI, these range from alterations of the blood flow to the kidneys, to blockage of the urinary tract, to severe infections (sepsis), to exposure to nephrotoxic agents, etc.

Whatever the triggering event, a growing body of literature shows that a tight glycemic control can reduce the incidence and severity of AKI as well as improve the outcomes of those patient who develop AKI (10). Although the mechanistic insights through which high glucose intensifies AKI have yet to be fully clarified, patients with a past episode of AKI are exposed to a greater risk of re-hospitalization and to new and repeated episodes of kidney injury, which facilitate progressive renal function decline, chronic kidney disease (CKD) and ultimately ESRD (11). Importantly, several non-diabetic comorbidities can also increase the risk for AKI; these include history of cancer, previous cardiac surgery and positivity to human immunodeficiency virus (HIV) (12). It is thus imperative to identify the key clinical characteristics of those AKI patients at higher risk for developing CKD in order to optimize their care.

## 2 AKI in diabetes

Patients with diabetes mellitus are commonly affected by a variety of comorbidities that increase their likelihood to develop AKI (12). Obesity, heart failure, hypertension, prior AKI episodes, CKD and even certain antihypertensive and antidiabetic medications are well known to be positively associated with the risk for AKI (13–15). Specifically, systemic hypertension is responsible for arterial and arteriolar hyalinosis, leading to vessel wall thickening, stenosis of the lumen and consequent reduced renal perfusion (16). Hyperglycemia itself promotes atherosclerosis, reduction of the vascular lumen and decreased renal perfusion, increasing the risk of myocardial infarction and stroke (17). Along, impaired cardiac function leads to decreased renal perfusion with subsequent reduction of the renal function. This turn of events is usually referred to as the “cardio-renal syndrome” (CRS) (18). Indeed, the incidence of AKI in diabetic patients is at least in part related to increased need for surgery, heavy drug regimen, frequent need to undergo diagnostic exams that can promote the development of contrast-induced AKI (CI-AKI). Moreover, these patients commonly suffer from bacterial infections involving the urinary tract and renal tissue, and are thus exposed to a high risk of developing sepsis or septic shock.

According to a recent study by Hapca et al., the incidence of AKI in patients with diabetes (in the absence of CDK) is 4.7 times higher compared to the non-diabetic population. These rates were obtained in a cohort including over 16,000 patients with and without diabetes. In the same study authors also found that patients with Type 2 Diabetes (T2D) show a steeper eGFR decline prior to AKI compared to non-diabetic individuals (19). When investigating AKI epidemiology in a large population retrieved from the UK General Practice Research Database, including 119,966 patients with T2D and 1,794,516 patients without diabetes, Girman et al. showed that, even after adjusting for age and specific comorbidities that are known risk factors for AKI (hypertension, obesity, prior AKI, CKD, congestive heart failure), the risk for AKI in the diabetic population was 2.2 times higher compared to the non-diabetic group (20). In a multicenter study investigating whether diabetes mellitus increases the risk of acute kidney injury (AKI) during sepsis and septic shock, Venot et al. reported that, although not associated with occurrence of AKI or need for renal replacement therapy (RRT), diabetes is an independent risk factor for persistent renal dysfunction in patients experiencing AKI (21).

Overall, despite the limited number observational studies published to date, type 2 diabetes (T2D) appears to be an independent risk factor for AKI, possibly by elevating the risk of cardiovascular complications (22).

To build an accurate tool for the estimation of individual patient’s risk for dialysis following cardiac surgery, Mehta et al. found diabetes as one of the several predictors of postoperative dialysis (23). In a sub-study conducted within the European Observational Sepsis Occurrence in Acutely ill Patients (SOAP), authors investigated the potential impact of insulin-treated diabetes on morbidity and mortality in ICU patients and found that insulin-treated diabetes was not an independent predictor of mortality (24).

To evaluate the association among pre-existing CKD with the risk of AKI, Hsu et al. compared a cohort of 1764 patients, who developed hospital-acquired AKI and were treated with dialysis, to 600,820 controls that did not develop AKI. Importantly, all cases and controls had outpatient estimated GFR measurements prior to hospitalization that were used to define baseline kidney function. Authors showed that patients with diabetes mellitus were at higher risk for AKI compared with their counterparts without diabetes mellitus even when in the same GFR category (25).

In conclusion a large body of evidence indicates underlying diabetes mellitus as an independent risk factor for AKI. In the next chapter we will analyze the molecular mechanisms through which hyperglycemia and established diabetes promote cellular dysfunction exposing the kidney to become more vulnerable to AKI.

## 3 Pathogenesis and molecular mechanisms of AKI in DM

As mentioned before, the abbreviation DKD is generally used to include all forms of renal damage in patients with diabetes, in fact, when a patient with diabetes shows the alteration of clinical signs such as proteinuria and eGFR, we can refer to the damage as DKD. Unfortunately, renal histopathology is still the gold standard to distinguish DN from NDRD within the generic umbrella term of DKD (26). In DN, specifically, the earliest histological signs include thickening of the glomerular basement membrane (GBM) and proliferation of the mesangial cells. In the more advanced stages of the disease, the progressive expansion of the mesangium causes the formation of matrix nodules, with mesangial cells arranged peripherally as a palisade (Kimmelstiel-Wilson nodules), compressing glomerular capillaries and causing microaneurysms. Hyalinosis of arteriolar walls, inflammatory infiltrate, interstitial fibrosis and tubular atrophy can also be present (for a complete description of the histological criteria used to classify DN refer to Tervaert et al. (27).

At the cellular level, DN and NDRD are driven by different underlying pathogenetic mechanisms. Herein, we will describe the main causes of AKI along with the effects of high glucose on the homeostasis of endothelial cells, podocytes and tubular cells, trying to explain how these cellular alterations contribute to increased susceptibility to AKI episodes.

### 3.1 Contrast-induced AKI in diabetes

AKI that occurs within 48-72h from intravascular administration of iodinated contrast media is known as contrast-induced AKI. CI-AKI is the third cause of hospital-acquired AKI (28) and some studies suggest that even a slight increase in blood glucose can intensify susceptibility to CI-AKI (29) thus, it is important to explore the causes that predispose patients with diabetes to develop this complication. A number of comorbidities that require more frequent radiological procedures compared to nondiabetic patients generally affects indeed diabetic patients. In these patients, injection of the contrast media can be responsible for alteration of the renal hemodynamic, reduced renal perfusion and subsequent hypoxia and ischemia (30–33). This hemodynamic effect is in part driven by a diminished ability of diabetic patients to counteract the renal vasoconstriction induced by the contrast media (34), also a direct toxicity of the iodinated contrast media in endothelial and tubular cells was described (35–37). Apoptosis of endothelial cells and reduced NO bioavailability leads to compromised vasodilation, while reabsorption of the water-soluble contrast media by proximal tubular cells can lead to morphological and functional changes, such vacuolization, increased ROS production, mitochondrial dysfunction with associated reduction in ATP synthesis, ER stress, disruption of the cell membrane integrity and cell death (38, 39). These events promote the release of damage-associated molecular patterns (DAMPs) and the activation of the innate immune response with subsequent release of cytokines (such as IL-1, IL-6, IL-18 and TNF-alfa) and chemokines (CCL2, CX3CL1 and CCL5) and further infiltration of inflammatory cells. The instauration of this chronic inflammatory status provides yet other mechanisms through which Diabetes intensifies CI-AKI (40, 41).

Lastly, high osmolality of the contrast media was proposed as a cause of nephrotoxicity in patients with compromised renal function and several studies investigated whether low-osmolar contrast media should be advised in the case of patients with diabetes or CKD. Results of a randomized, double blind, prospective, multicentre study aiming to asses nephrotoxicity in patients at risk for CI-AKI showed that the administration of the iso-osmolar non-ionic iodixanol is protective in this cohort when compared to the low-osmolar, non-ionic iohexol (42).

### 3.2 Sepsis and AKI in diabetes

Sepsis is the most common cause of AKI in critically ill patients and is characterized by an intense systemic inflammatory response to infection which can lead to multiple organ failure (43, 44). Whether diabetes correlates with more severe infections and increased risk of AKI is still controversial (22). Clinical experience suggests that DKD, a condition notoriously characterized by immune dysfunction, metabolic imbalances and hyperglycemia, could increase the susceptibility to acute infections and may also impair the host immune response resulting in worse outcomes (22, 45).

Hyperglycemia has been linked with adverse outcomes in acute settings such as acute coronary syndrome and brain injury (46, 47). The metabolic alterations induced by high glucose are responsible for increased mitochondrial ROS production, the release of free fat acids and the inactivation of two critical antioxidant enzymes, eNOS and prostacyclin synthase (22, 46). These events impair endothelial function in both macro- and microvascular beds, disrupting the homeostasis in all organs, including the kidney (48). The treatment of endothelial dysfunction, along with the development of new approaches able to restore the metabolic pathways altered by hyperglycemia could prevent vascular dysfunction and decrease susceptibility to AKI.

Several studies analyzed the importance of glucose control in intensive care units ‘patients defying the incidence of AKI. Thomas et al. performed a systematic review and meta-analysis across clinical studies comparing ‘conventional’ vs ‘intensive’ insulin therapy in critically ill patients, evaluating the risk of AKI (49). Interestingly, their analysis indicated a strong 38% decrease in the incidence of AKI in intensified insulin treated- patients for overall studies.

Recently, Venot et al. analyzed the incidence of AKI and the need of renal replacement therapy in both diabetic and nondiabetic patients with severe sepsis or septic shock enrolled in a prospective database by 12 intensive care units (ICUs) (21). This study showed that diabetes is an independent risk factor for chronic renal dysfunction in patients who experienced septic AKI.

A multi-center, open label, randomized controlled trial of glucose management, the NICE-SUGAR study (NCT00220987), compared the effects of strict insulin regimen, maintaining blood glucose between 4.5-6.0 mmol/L versus a liberal approach with glucose levels between 8.0 - 10.0 mmol/L (50, 51). Interestingly, the risk of death was greater in the intensive glycemic control arm due to more frequent hypoglycemic events. The incidence of AKI however did not differ between the two groups.

Recently, the Surviving Sepsis Campaign severe sepsis and septic shock recommended a glucose control protocol with blood glucose levels ≤180 mg/dL rather than ≤110 mg/dL (52). Thus, AKI incidence may be reduced without further aggravating mortality.

Another aspect that needs to be discussed is the etiopathogenesis of AKI in course of sepsis and how diabetes could exacerbate the principal mechanisms involved. Numerous experimental investigations have underlined the central role of the overwhelming inflammatory response and renal cells dysfunction in this setting (43, 44, 53–55). Tubular damage is evidenced on biopsy by exfoliation, vacuolization of tubular cells, loss of brush border and tubular necrosis (56). Several studies have also shown overexpression of KIM-1, which is a marker of proximal tubule damage in these patients (57, 58). At the level of the glomerular capillaries, on the other hand, an infiltrate of polymorphonuclear and monocyte-macrophages is generally present, along with deposition of fibrin in the lumen (59). The peritubular capillaries and the interstitial compartment may present an intense inflammatory infiltrate. Furthermore, the premature deposition of collagen fibers has recently been highlighted in the tubulointerstitial space, along peritubular capillaries, underlying how parenchymal fibrosis could occur in early stages of renal damage (60–62).

During sepsis, an intense inflammatory response occurs, cytokines and chemokines are released, complement is activated and ROS production is increased (63, 64). Proinflammatory cytokines activate the endothelium and promote the expression of leukocyte adhesion molecules and of the Tissue Factor (TF) favoring the formation of microthrombi within renal capillaries (65–67). An important role is played by the activation of the complement that alters vascular homeostasis and increases leukocyte-endothelium adhesion, causing a reduction in renal perfusion and damage to ischemic/reperfusion (I/R) (43, 68). C5a is the most important pro-inflammatory mediator as it is able to stimulate the production of TNF-α, IL-6 and cytokines monocyte chemoattrant protein-1 (69). C5b-9 activates endothelial nuclear factor-кB increasing leukocyte-endothelial adherence by overexpressing E-selectin, P-selectin, vascular cell adhesion molecule 1 and intercellular adhesion molecule 1 (70). Recently, Feketeova et al. showed an exacerbated inflammatory response to bacterial endotoxin in diabetic mice, characterized by increased TNF production in the spleen and responsible for systemic inflammation (71); it remains unclear whether chronic diabetes, which is more clinically relevant, affects this response.

A clear answer to which molecular mechanisms involved in diabetes impact the onset of sepsis and the incidence of AKI require a translational approach based on both preclinical and *in vitro* studies in association with epidemiological research. Further studies are therefore needed to better define the questionable association between diabetes, sepsis and AKI.

### 3.3 Drug-induced AKI in diabetes

Drug-induced AKI accounts for 20% of all AKI episodes (72, 73); the specific mechanisms of pathogenesis vary according to the drug or drug combination administered and besides direct nephrotoxicity, several medications can promote kidney damage indirectly, through a reduction in the renal perfusion or an alteration of the renal hemodynamics. The medications more frequently related to drug-induced AKI in patients with diabetes include: aminoglycosides (eg. Gentamicin), non-steroidal anti-inflammatory drugs (NSAIDs), angiotensin-converting enzyme inhibitors (ACEIs), angiotensin receptor blockers (ARBs), lipid-lowering agents (statins), diuretics and Rifampicin (74, 75). Lapi et al. identified the triple therapy combination consisting of ACEIs/ARBs, NSAIDs and diuretics as being particularly associated with an increased risk of admission for AKI as a primary diagnosis (76). Among the most widely prescribed medications for the treatment of diabetes mellitus, metformin and SGLT-2 inhibitors deserve a separate discussion. Metformin is an oral antidiabetic drug that belongs to the class of biguanides and is the first-line therapy for type 2 diabetes due to its low incidence of micro and macrovascular events (77). Metformin is able to increase the uptake and utilization of glucose by insulin-sensitive peripheral tissues and to counteract insulin-resistance *via* reduction of hepatic glucose production (gluconeogenesis). With respect to its mechanism of action, metformin activates the AMP-activated protein kinase (AMPK) signaling, a pathway with a key role in the regulation of intracellular and whole-body energy metabolism (78). A recent study showed that metformin could selectively inhibit the mitochondrial glycerol-3-phosphate dehydrogenase (GPD2) and promote accumulation of cytosolic NADH. This accumulation results in reduced conversion of lactate (one of the substrates of gluconeogenesis) into pyruvate, promoting the accumulation of lactate in the bloodstream (79, 80). Of note, lactic acidosis is a well know complication of therapeutic regimens that include biguanides, it occurs when lactic acid production exceeds lactic acid clearance. Lactate is a metabolic end product of anaerobic glycolysis and biguanides such as metformin can cause lactic acidosis by blocking aerobic glycolysis and increasing anaerobic glycolysis through inhibition of the mitochondrial respiratory chain, particularly of the mitochondrial complex I (81, 82). It is important to point out that lactic acidosis is mostly a risk in patients with organ dysfunction, such as patients with congestive heart failure or renal impairment. Despite the above-mentioned risks, metformin is a valuable compound with many benefits, as it was shown to also effectively increase fatty acid oxidation, decrease the intestinal absorbance of glucose, and delay the gastric emptying thus reducing appetite and stimulating weight loss.

Inhibitors of the sodium-glucose cotransporter 2 (SGLT2) represent a novel class of drugs increasingly prescribed for both diabetic and non-diabetic patients for their protective effects on the kidney and the heart (83–85). The SGLT2 cotransporter is expressed in the proximal tubule and works by reabsorbing sodium along with the almost totality of the glucose filtered by the glomerulus (90%) (86). During hyperglycemia, the upregulation of SGLT2 enhances the tubular reabsorption of glucose but persistently high levels of glucose are associated to hypertrophy and hyperplasia of mesangial and tubular cells even in the early stages of the disease (87). Of note, the reabsorption of glucose *via* SGLT2 is coupled to the reabsorption of sodium and this generates an influx of liquids and a diminished NaCl and fluid delivery to the macula densa, altering the tubuloglomerular feedback and stimulating glomerular hyperfiltration. SGLT2 inhibitors prevent glucose and sodium reabsorption, reduce the workload of the tubular cells and restore the tubuloglomerular feedback (88). It was also reported that SGLT2 inhibitors can suppress the plasma levels of TNF receptor 1, IL-6, matrix metalloproteinase 7 (MMP7) and fibronectin 1, displaying additional anti-inflammatory and anti-fibrotic properties (89).

Finally, this novel class of drugs seems to reduce oxidative stress, a characteristic that could be responsible for increased protection against cardiovascular events (90). Despite these widely acknowledged protective effects, some studies highlighted an early but reversible reduction of the GFR in patients treated with SGLT2 inhibitors, followed by a preserved GFR in the long term (91). This early decrease of the GFR has been shown to increase the risk of AKI, especially in more susceptible individuals such as those with impaired renal function (92) although several others studies did not find this correlation (93–95).

### 3.4 AKI and endothelial dysfunction in the diabetic patient

Under normal conditions, the endothelial cell layer that lines the entire circulatory system finely regulates the release of vasoactive substances that in turn maintain the vascular tone and regulate platelet and leukocyte adhesion to the vascular surface, preventing atherosclerosis. Alterations of the vascular permeability are important drivers of proteinuria and can be detected in AKI as well as in CKD. Over the past, dysregulation of specific angiogenic factors, inflammatory and adhesion molecules was discovered in many pathological conditions. The master regulator of endothelial homeostasis is nitric oxide (NO) whose main functions are to i) promote vasodilation *via* signaling to the surrounding smoot muscle cells, ii) inhibit coagulation and thrombosis and iii) suppress vascular inflammation.

Diabetic patients with endothelial damage show reduced bioavailability of endothelial NO (96) and sustained hyperglycemia is associated with injury to the glomerular afferent and efferent arterioles along with damage to the interstitial peritubular capillary vessels it supplies (26, 97); additionally, the compresence of dyslipidemia, hypertension and increased plasma free fatty acids significantly increases the risk of atherosclerosis and cardiovascular complications in these patients.

The progressive vascular rarefaction due to overproduction of reactive oxygen species (ROS) and endothelial cells damage in the kidney, leads to hypoxia and compromised proximal tubule function. Under these circumstances, a diabetic state can aggravate the hypoxic state during episodes of AKI (98, 99), explaining why ischemic-reperfusion injury (IRI), sepsis, surgery or administration of contrast agents, exposes diabetic patients to a greater risk of AKI compared to non-diabetic patients. Preservation of peritubular capillary endothelial integrity and increased activation of pericytes may be critical to recovery from post-ischemic AKI (100) and diabetes leads to the loss of endothelial-pericyte interaction, driving the progression of fibrosis (101). These changes also occur during AKI with the activation and proliferation of pericytes and their transformation into myofibroblasts, which drive fibrosis (102), by Pericyit-to a-Myofibroblast trans-differentiation, regulating the process of vascular rarefaction of the peritubular capillaries (61, 103).

At the cellular and subcellular level, persistently elevated blood glucose levels per se, induce the formation of advanced glycation end-products (AGEs) that can accumulate in endothelial cells promoting monocyte adhesion and migration *via* activation of NF-κB with subsequent transcription of proinflammatory cytokines (104, 105). In patients with diabetes, NF-κB was also responsible for increased levels of endothelin-1 (ET-1) (106), one of the most potent vasoconstrictors known. *In vitro*, persistently elevated blood glucose levels were shown to cause endothelial cells apoptosis through the activation of NF-кB and c-Jun NH2-terminal pathways (107).

More recently, novel insights in the field of vascular biology are coming from the study of specific cell populations/cell structures, such as pericytes, endothelial glycocalyx and capillary fenestrations. Pericytes are specialized cells that provide support and stability to small vessels (arterioles, capillaries and post-capillary venules). Pericytes are particularly abundant within the capillary branches of the central nervous system and the retina, but are also found in the renal tubular system. Morphologically, the pericyte has extensive cytoplasmic processes able to engulf multiple endothelial cells. Although the molecular mechanism is still largely unclear, the close interaction between pericytes and endothelial cells appears crucial to maintain vascular homeostasis, specifically proper filtration. Importantly, it was recently shown that diabetes-associated microvascular complications could be at least in part due to pericyte dropout and disruption of the pericyte-endothelial cell crosstalk (108). Pericyte dropout is a hallmark of diabetic retinopathy (109), also within the kidney, loss of podocytes (pericytes-like cells) is a hallmark of diabetic nephropathy and contributes to proteinuria. The endothelial glycocalyx is a polyanionic gel composed of proteoglycans, glycoproteins and glycosaminoglycans. This gel coats the luminal surface of all endothelial cells and, when damaged, can lead to increased vascular permeability with escape of water and macromolecules (110). Damage of the glycocalyx ultrastructure was reported, among others, in conditions such as AKI, CKD, and diabetes (111, 112). Finally, decreased filtration in diabetic glomeruli is at least in part related to ultrastructural changes including decreased fenestration density within glomerular endothelial cells (113).

### 3.5 AKI, podocyte injury and depletion in DKD

Podocytes are highly specialized epithelial cells that line the outer surface of glomerular capillaries *via* attachment to the glomerular basement membrane (GBM). Podocytes are actively involved in the regulation of glomerular permeability and participate in the synthesis of GBM proteins (114). These complex functions depend on the peculiar cytoarchitecture of the podocyte, characterized by several cytoplasmic extensions that interdigitate around the glomerular capillaries. These cytoplasmic extensions are known as pedicles (or foot processes) and the narrow slit between adjacent pedicles is known as slit diaphragm (SD), crucial to regulate the filtration of plasma proteins from the glomerular capillaries into the proximal convoluted tubule (115). The structural and functional integrity of the slit diaphragm is provided by a specific set of proteins, including, among others, Nephrin and Neph1 that form a flexible filtration layer among adjacent foot processes. Any damage occurring at the level of the SD proteins can ultimately lead to cytoskeleton disorganization, podocyte effacement and proteinuria.

Several studies have shown that patients with DKD display a reduced density of podocytes and a compromised molecular architecture of the SD (116). As previously mentioned, podocytes are pericytes-like cells that line glomerular capillaries preventing protein leakage into the tubular compartment. Similarly to the damage caused by high glucose on pericytes, podocytes exposed to hyperglycemia show a progressive effacement of their foot processes and ultimately undergo apoptosis. Podocyte loss thus rapidly causes albuminuria, and increased albumin uptake in turns accelerates tubular epithelial cell injury and interstitial fibrosis; importantly, podocytes have a low capacity to regenerate, thus podocyte loss can lead to a permanent damage (117, 118). *In vitro*, increased levels of glucose were shown to stimulate intracellular ROS production and subsequent activation of the p38 MAPK and caspase 3 pro-apoptotic pathways, leading to podocyte loss (119). With respect to the role of podocyte depletion and tubular fibrosis, Munkonda et al. demonstrated that, during diabetes, injured podocytes can secrete small vesicles that then travel to the tubular compartment and activate p38 mitogen-activated protein kinase (MAPK) and CD36 signaling inducing pro-fibrotic responses with increased expression of the ECM proteins fibronectin and Collagen IV (120).

Several experimental evidences also point to abnormal activation of the slit diaphragm-associated protein transient receptor potential canonical (TRPC6) calcium channel among the causes of high glucose-induced podocytes depletion and proteinuria (121, 122). It has been suggested that increased renin-angiotensin system (RAS) activation and elevated levels of angiotensin II, frequently observed in patients with diabetes, lead to TRPC6 activation with subsequent increased intracellular calcium (Ca2+) influx and increased podocytes apoptosis (123); moreover, deletion of TRPC6 in diabetic mice preserves renal histology and prevents albuminuria (122).

Increased angiotensin II, along with increased glycated albumin, have also been implicated in the reduction of nephrin expression (124), and mutations of nephrin are associated to the congenital nephrotic syndrome of the Finnish type (CNF), characterized by heavy proteinuria (125). Aside from its important structural functions, nephrin is also implicated in the molecular mechanism of insulin-mediated glucose uptake in podocytes (126).

It has been shown that the severity and duration of AKI episodes can affect the susceptibility to CKD and that AKI-CKD transition is encouraged when the aberrant activation of signaling pathways, such as RAS activation, is not corrected. RAS signaling was shown to be induced *in vivo* following systemic administration of LPS (127) (50) thus RAS activation is likely to contribute to the maladaptive repair mechanisms that follow AKI episodes and that are ultimately responsible for nephron loss, vascular rarefaction, chronic inflammation and fibrosis.

Among the intracellular signaling pathways alterations induced by hyperglycemia, inhibition of autophagy was also discovered in podocytes (128). Autophagy is an evolutionary conserved mechanism that cells use to replace damaged cellular components with new ones, thus it is particularly important in those cell types with limited capacity to proliferate and regenerate as it prevents the accumulation of toxic intracellular products such as protein aggregates and oxidized lipids (129). Autophagy also allows the maintenance of cellular homeostasis following specific stress events, for instance during hypoxia or oxidative stress, preventing cellular starvation through the catabolism and recycling of intracellular components such as proteins and organelles.

A wide-range of events can trigger autophagy during AKI, ROS production and oxidative stress in particular and can lead to mitochondrial dysfunction and autophagy of mitochondria (mitophagy) in the kidney, where these organelles are very abundant. Experimental evidence suggests that mitophagy provides cytoprotection and prevents renal fibrosis; interestingly, reduced autophagic activity was observed *in vitro* and *in vivo* in podocytes exposed to high glucose conditions (128), as well as in patients with massive proteinuria (130), suggesting that insufficient autophagy within podocytes can lead to podocyte injury in DKD. Other studies suggested that the impairment of autophagy in podocytes might be due to AGEs accumulation and subsequent mTOR hyperactivation, since pharmacological inhibition of mTOR could restore the autophagic flux even in the presence of hyperglycemia (131, 132). The specific molecular mechanisms through which insufficient autophagy in podocytes leads to DKD are still under investigation however it was recently shown that a chronically high levels of angiotensin-II can inhibit the autophagy flux *via* the activation of calpains in podocytes. Calpains are calcium-activated proteases; using *in silico* prediction tools, these proteases are predicted to cleave proteins that are crucial to podocyte viability, such as nephrin and podocin, along with proteins that regulate the autophagic flux such as Beclin-1 and ATG5 (133).

### 3.6 AKI and proximal tubular injury in DKD

The typical manifestations of DKD are proteinuria and altered eGFR, these clinical signs encouraged researchers to focus on the glomerulus in order to identify the causes of DKD progression. Today we know that 20% of patients with DKD can progress to ESRD despite absence of proteinuria (134) and that tubular hypertrophy is a very early sign of DKD as it can be observed in patients with normoalbuminuria within a few days from hyperglycemia (135). It has recently been suggested that tubular dysfunction might be the “*primum movens*” in DKD and that glomerular hyperfiltration is only a consequence of increased proximal tubule reabsorption and compromised tubuloglomerular (TGF) feedback due to insufficient sensitivity of the tubule to regulatory signals (136).

Indeed, the reabsorption activity of the renal tubule is energetically demanding, and when the oxygen supply does not meet the oxygen demand, renal ischemic injury due to hypoxia can occur (137). Under high glucose conditions, excess glucose is actively transported (along with Na+) into the proximal tubular epithelial cells through the sodium-dependent glucose transporters 2 (SGLT-2). Although this transport does not require energy, it relies on the maintenance of an electrochemical gradient, ensured by the activity of the Na+/K+ ATPase pump. Thus, under hyperglycemic conditions, the activity of the Na+/K+ ATPase pump is increased and more oxygen is consumed, suggesting that diabetes increases the sensitivity of tubular cells to ischemia reperfusion injury (IRI) and renders tubular cells more prone to apoptosis. Accordingly, the beneficial effects of the therapeutic regimens with SGLT2 inhibitors, are not linked exclusively to the promotion of glycosuria and natriuresis, but also to the reduced oxygen consumption that derives from the inhibition of glucose and sodium uptake. Furthermore, although the FDA issued initial warnings suggesting that Canaglifozin and Dapaglifozin administration in particular could promote AKI, later studies proved that their use, was not associated to increased risk for AKI compared with other glucose-lowering agents (93).

Among the pathogenic mechanisms that promote tubular injury in DKD, mitochondrial dysfunction can drive the loss of tubular integrity. Mitochondria are very abundant in proximal tubular epithelial cells, as high concentrations of ATP are required to ensure appropriate tubular reabsorption. When glomerular hyperfiltration occurs, tubular reabsorption is exacerbated, in this state intensive ATP production within mitochondria in induced. Excessive ATP production however is associated to increased oxidative stress and consumption of the cellular antioxidant capacity. Mitochondrial dysfunction can trigger cell death activation, and studies *in vitro* and *in vivo* have shown that proximal tubular cells cultured in high glucose and diabetic mice respectively, were more susceptible to apoptosis and caspase activation following ATP consumption and hypoxia and that suppression of p53 could have beneficial effects *in vitro* by diminishing high-glucose sensitivity and *in vivo* by reducing the ischemic AKI in diabetic mice (138). Thus, the restoration of mitochondrial homeostasis could provide important benefits to attenuate AKI sensitivity in patients with diabetes and in general to improve the current therapeutic strategies against DKD.

Aside from hypoxia and mitochondrial dysfunction, a chronic hyperglycemic state in the kidney causes inflammation, activation of the intrarenal innate immune response, hyperplasia and fibrosis (139). Indeed, patients with diabetes show high levels of pro-inflammatory cytokines such as tissue necrosis factor (TNF)-α, interleukin (IL)-1 and IL-6 compared to non-diabetic individuals and this higher baseline inflammatory state can worsen the clinical outcomes of patients with diabetes that undergo acute kidney damage (40, 140, 141). In addition, diabetic kidneys present higher expression of chemoattractant cytokines and this promotes the intrarenal infiltration and accumulation of macrophages (142). Traditionally, two different subtypes of macrophages can be distinguished: M1 and M2. The M1 phenotype is pro-inflammatory and its recruitment occurs over the early stages of renal damage during an AKI episode. The M2 phenotype instead promotes the replication of renal tubular cells allowing tissue remodeling following injury. A positive correlation between the M1/M2 activation state and renal damage was found in DN (143).

Stress of the endoplasmic reticulum (ER) due to altered cellular redox and protein misfolding is also implicated in the pathogenesis of both diabetes, AKI, and DKD (144–146) and is an important driver of tubulointerstitial fibrosis in diabetes. Using an elegant transgenic mice model where diabetes was induced by Streptozotocin injection, Sharma et al. showed that increased expression of the enzyme myo-inositol Oxygenase (MIOX) during hyperglycemia is associated with worse renal function and increased oxidant/ER stress. In transgenic MIOX-KO mice instead, MIOX gene disruption is protective against oxidant stress (147).

Finally, a novel mechanism of tubular injury in DKD is linked to the tubular accumulation of ubiquitinated proteins, specifically proteins ubiquitinated *via* lysine 63 polyubiquitin chains. This accumulation was shown to impair autophagy and activate apoptosis (148, 149) and its inhibition can prevent EMT of tubular cells grown in high-glucose as well as renal fibrosis in STZ-treated DBA/2J mice (150). Thus, the deregulation of autophagy does not only affect podocytes but also tubular cells within the kidney.

**Figure 1:**
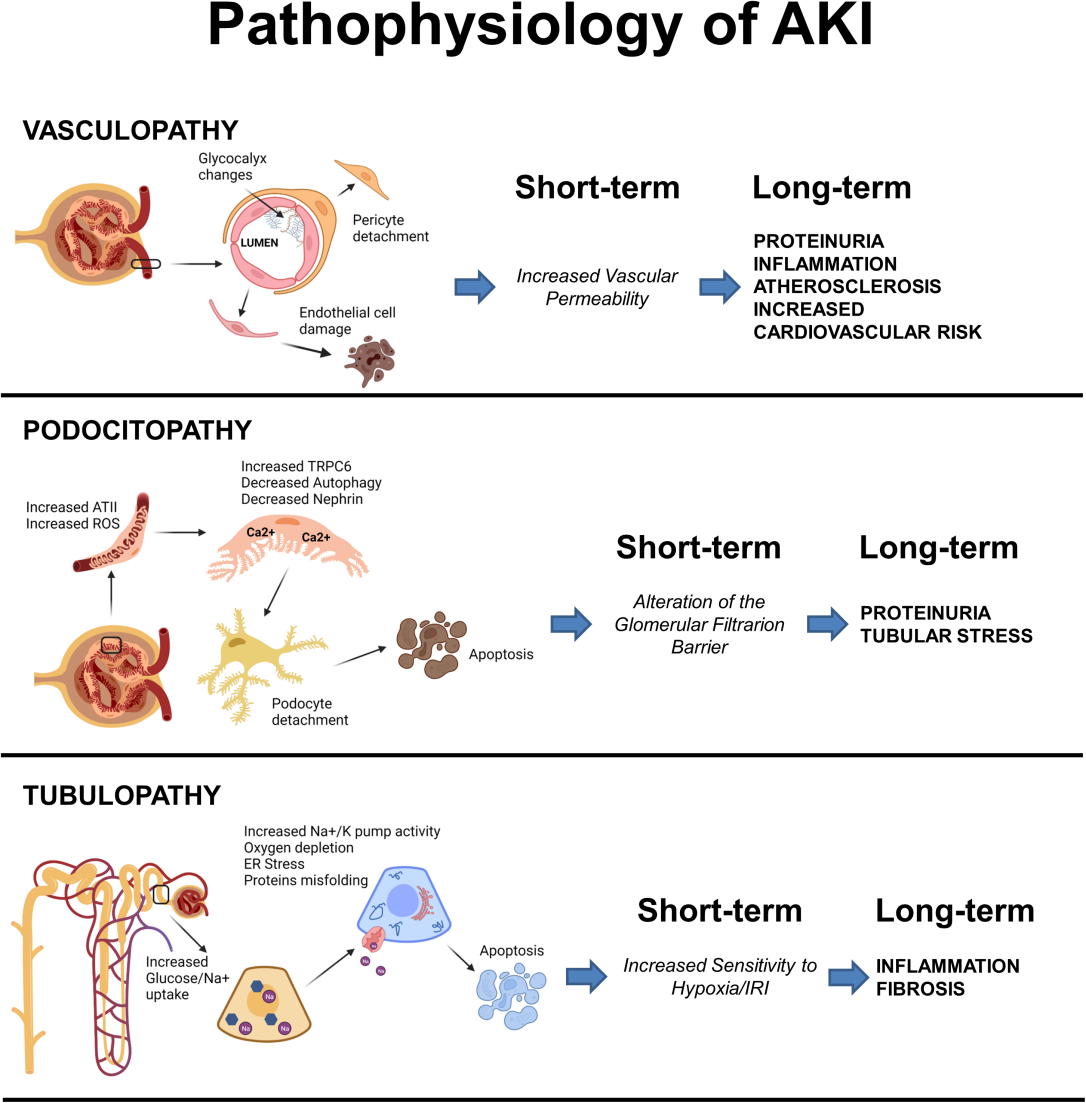
Schematic illustration of the main events driving vasculopathy, podocitopathy and tubulopathy in AKI and Diabetes. Created with BioRender.com.

In conclusion, diabetes increases the susceptibility to AKI through the instauration of multiple maladaptive repair mechanisms that involve all renal compartments and all renal cell types, a schematic illustration of the main drivers of vasculopathy, podocitopathy and tubulopathy can be viewed in **Figure 1**. See **Table 1** for a comprehensive summary of the experimental evidences that support these hypotheses and that were reported in this section.

**Table 1:** Signaling pathways, target molecules and candidate treatments against maladaptive repair in AKI and diabetes.

Renal Compartment	Cell Type	Signaling Pathway	Histological signs	Proposed Targets	Treatments/Compounds	Type of Study	Reference
**Vascular**	Endothelial cells	Inflammation	Atherosclerosis	Nitric Oxide, AGEs	Statins, HMG CoA reductase inhibitors	*In vivo* pre-clinical studies	(151, 152)
**Vascular**	Endothelial cells	Hypoxia, IR injury	Vascular Rarefaction	ROS	Antioxidant supplementation	*In vitro* sutdies, *in vivo* pre-clinical studies	(153)
**Vascular**	Endothelial cells	Apoptosis	Loss of renal mass, Collagen deposition	ET-1	ETA-blocking agents	*In vitro* sutdies, *in vivo* pre-clinical studies	(154)
**Vascular**	Pericytes	Fibrosis	Loss of Endothelial-Pericyte Interactions	Complement system	C1-inhibitor (C1-INH)	*In vitro* sutdies, *in vivo* pre-clinical studies	(154)
**Glomerular**	Podocytes	Apoptosis	Podocyte Loss	SGLT2	SGLT-2 inhibitors	*In vitro* sutdies, *in vivo* pre-clinical studies, Human biopsies	(155)
**Glomerular**	Podocytes	Apoptosis	Podocyte Loss	TRPC6	TRPC6 silencing	*In vivo* pre-clinical studies	(122)
**Glomerular**	Podocytes	RAS Activation, Apoptosis	Reduced Nephrin	Angiotensin II, Glycated Albumin	ACEi, ARBs	Human biopsies	(124)
**Glomerular**	Podocytes	Impaired Autophagy	Reduced autophagic vacuoles	AGEs, mTOR	mTOR inhibition	*In vitro* sutdies, *in vivo* pre-clinical studies, Human biopsies	(131)
**Tubular**	Tubular Cells	Hypoxia, IR injury	Increased HIF1-a	ROS	SGLT-2 inhibitors	*In vitro* sutdies, *in vivo* pre-clinical studies	(156)
**Tubular**	Tubular Cells	Hypoxia, Apoptosis, Ischemia	Severe tubular damage	ROS, p53	p53 inhibition, Pifithrin-α	*In vitro* studies, *in vivo* pre-clinical studies	(138)
**Tubular**	Tubular Cells	Inflammation/Fibrosis	Infiltration of macrophages	TNF-a, IL-1; IL-6	SGLT-2 inhibitors	*in silico* models; *in vitro* studies	(88)
**Tubular**	Tubular Cells	Impaired Autophagy/Fibrosis	Epitelial to Mesenchimal Trasition	UBE2v1	NSC697923	*In vivo* pre-clinical studies	(150)

## 4 Novel frontiers in the prevention of AKI in diabetes

Numerous studies have underlined the efficacy of antioxidants in preventing I/R damage, such as superoxide dismutase (SOD), catalase, allopurinol, vitamin E (157). As we have previously illustrated for the purpose of the pathogenetic mechanisms, in the course of AKI, and especially in diabetic patients, there is an upregulation of the complement. For this reason, an inhibition of the complement pathway at different levels may reduce the damage. The C3 convertase inhibitor and antibody against C5 factor significantly attenuate complement activation (158, 159); C1-inhibitor (C1-INH), able to arrest the progression of fibrosis and the accelerated renal aging, also seems to have a key role (61, 103).

Recently, stem cell-based therapy showed encouraging results in renal diseases, including diabetic nephropathy (160). It has been well accepted that stem cells participate in repair processes in damaged kidney mostly *via* paracrine action and immunomodulation (161, 162). Stem cells can release several immunomodulatory factors with a wide range of immunomodulatory effects that could trigger intracellular signaling in dysfunctional renal cells and promote regeneration (161). Lee et al. showed that multipotent stromal cells from human bone marrow could provide a potential therapy to repair pancreatic islets and renal glomeruli in diabetic mice (163). In accordance, another study demonstrated that mesenchymal stem cells administration in type 1 diabetic rats prevented the development of albuminuria and reduced the loss of podocytes by increasing BMP-7 secretion, reducing renal damage (164).

Several cell-fate tracking studies showed that human adult renal progenitor cells (ARPCs) could revert renal damage dedifferentiating in adult tubular epithelial cells (165, 166). In addition, these cells contribute to renal recovery by secreting regenerative molecules that enhance the tubular repair mechanisms (167) and prevent endothelial dysfunction during AKI (55). Therefore, these cells could directly and indirectly drive the renal repair process and could offer a new therapeutic strategy for future clinical applications.

## 5 Conclusion

In summary, there is a tight interplay between the molecular pathways that promote AKI and diabetes, thus diabetes is undoubtedly an independent risk factor for AKI episodes. Patients with diabetes are more susceptible to hypoperfusion, which aggravates the hypoxic state during AKI. On the other hand, AKI is itself a major risk factor for DKD.

The further understanding of the specific pathogenetic mechanisms and biomarkers of progression will allow developing and evaluating future therapeutic strategies capable to prevent, slow down or reverse the progression of acute and chronic kidney damage in diabetes.

## Author contributions

BI, FC and PP planned the review and wrote the manuscript. AS, DSA, SL, DT, MF and CA participated in the design of the review and critically contributed to draft the manuscript. LG, GC and GS participated in the coordination of the manuscript and critically reviewed it. All authors contributed to the article and approved the submitted version. 
